# Opportunities and challenges of large-scale screening for atrial fibrillation

**DOI:** 10.1007/s00399-017-0550-y

**Published:** 2018-01-08

**Authors:** Matthias Daniel Zink, Nikolaus Marx, Harry J. G. M. Crijns, Ulrich Schotten

**Affiliations:** 1 Department of Physiology, Cardiovascular Research Institute Maastricht, Maastricht University, Universiteitssingel 50, 6229 ER Maastricht, The Netherlands; 2 Department of Cardiology, Pneumology, Angiology and Intensive Care Medicine, University Hospital RWTH Aachen, Aachen, Germany; 3 Department of Cardiology, Cardiovascular Research Institute Maastricht, Maastricht University Medical Centre, Maastricht, The Netherlands; 4 Maastricht University, P.O. Box 616, 6200 MD Maastricht, The Netherlands

**Keywords:** Cardiac arrhythmia, Subclinical, Screening, Single-lead ECG, Electrocardiography, Herzrhythmusstörung, Asymptomatisch, Screening, 1-Kanal-EKG, Elektrokardiographie

## Abstract

Atrial fibrillation (AF) is a common arrhythmia and is highly associated with stroke and cardiovascular morbidity. As many AF episodes remain subclinical (SCAF), large-scale AF screening is considered a desirable approach for the treatment and prevention of cardioembolic stroke. Newly available single-lead ECG devices have provided promising results in the diagnosis of SCAF and treatment by nonvitamin K antagonist drugs appears to be safe and effective. Nevertheless, a further gain in knowledge is needed to clarify the different types of AF. This may help to define how or if a patient should be treated in the context of outcome and cost effectiveness. This review summarizes the results of recent research in this field and focuses on single-lead, discontinuous single time-point, community-based comprehensive-screening-based AF management. We want to conclude that progress in ECG diagnosis and anticoagulation therapy has prepared the ground to establish large-scale AF screening. The remaining question, however, is which patients should be screened and what therapy should be initiated in case of AF.

## Zusammenfassung

Vorhofflimmern (AF, „atrial fibrillation“) ist eine weitverbreitete Herzrhythmusstörung und häufig mit Schlaganfall und kardiovaskulärer Morbidität vergesellschaftet. Viele AF-Episoden verlaufen asymptomatisch (SCAF), sind aber trotzdem mit einem erhöhten Schlaganfallrisiko assoziiert. Daher erscheint ein AF-Massenscreening als erstrebenswerter Ansatz zur Behandlung und zur Prävention von Schlaganfällen. Neu verfügbare 1‑Kanal-EKG-Geräte zeigen in der Erkennung von asymptomatischem AF vielversprechende Ergebnisse. Zudem könnten die Nicht-Vitamin-K-Antagonisten Vorteile im Blutungsrisiko gegenüber den Vitamin-K-Antagonisten aufweisen. Nichtsdestotrotz ist weiteres Wissen über verschiedenen Stadien von AF notwendig. Dies kann bei der Entscheidung helfen, ob und wie ein Patient im Sinne von Outcome und Kosteneffektivität behandelt werden kann. Dieser Übersichtsartikel fasst aktuelle Ergebnisse von Studien auf diesem Gebiet zusammen und konzentriert sich auf bevölkerungsweite 1‑Kanal-EKG-Messungen für ein screeningbasiertes AF-Management. Wir schlussfolgern, dass Fortschritte in der EKG-Diagnostik und in der Antikoagulationstherapie die Grundlage für ein bevölkerungsweites AF-Screening gelegt haben. Die verbleibenden Fragen sind daher, bei welchen Patienten nach AF gesucht und welche Behandlung im Falle eines AF begonnen werden soll.

## Introduction

Atrial fibrillation (AF) is highly associated with stroke, heart failure, sudden death, cardiovascular morbidity, and reduced quality of life [12]. The majority of all strokes are ischemic [28] and are accompanied by a high coincident diagnosis of AF [9, 29]; in addition, one quarter of ischemic strokes are considered as embolic stroke of unknown source (ESUS). As many AF episodes remain asymptomatic, a stroke is the first clinical manifestation of AF for a quarter of all patients. Thus, early detection and correct treatment of AF may importantly help to prevent stroke.

Considering the requirements for mass screening stipulated by the WHO [27], there is as yet not enough evidence to implement such screening and align it in structured care. In addition, more evidence is needed regarding who shall be screened and how AF should be subsequently treated to determine cost effectiveness.

## Rationale for screening

By the definition of the WHO by Wilson and Jungner [27], four main aspects, which include knowledge of disease, knowledge of test, knowledge of treatment, and cost considerations, must be fulfilled for a successful mass screening approach.

### Knowledge of disease

AF is the most common arrhythmia with increasing prevalence by age and a long latent phase of structural atrial remodeling [5]. About one third of all patients with new-onset AF suffer from typical symptoms like palpitations, while the remainder are asymptomatic or suffer from atypical symptoms like fatigue [17, 23]. The risk for stroke is higher for permanent compared to paroxysmal AF [26] but present in all stages of AF and does not necessarily correlate with the contemporary occurrence of AF [3] or symptoms. Despite considerable efforts, the exact electrophysiological mechanisms leading to AF remain uncertain. Focal ectopy and re-entry are accepted mechanistic explanations for AF induction and perpetuation but current research endeavors hardly relate these mechanisms to patterns of AF episodes. In this sense, screening for AF has also the potential to deepen our understanding of AF pathophysiology.

### Knowledge of test

Guidelines exist how to screen for SCAF and to subsequently diagnose clinical AF using standard electrocardiography techniques. However, diagnosing clinical AF after detecting SCAF is a challenge because AF episodes often self-terminate precluding capture on standard ECG. To identify patients at risk for AF, current guidelines recommend a thorough clinical history for the determination of risk factors as well as a physical examination including auscultation and pulse palpation [10, 12]. If AF is suspected, a 12-lead ECG needs to be recorded to confirm the diagnosis. In particular, clinical suspicion of paroxysmal AF may therefore lead to continuous or repeated ECG recordings using event recorders or Holter ECGs.

### Treatment of disease

The AF event rate was significantly lowered by an improved risk factor management like the usage of angiotensin-converting enzyme (ACE) inhibitors or angiotensin receptor blockers (ARBs) for hypertension control. However, despite technological advances in catheter ablation and numerous attempts of development of new antiarrhythmic compounds, only incremental improvements in rhythm control have been achieved [13]. Therefore, the challenge of treatment is to install effective stroke and risk factor prevention, rather than establish effective rhythm control. A reason for these limited advances in rhythm control may be that AF patients are frequently diagnosed and treated at relatively late stages of their disease. However, it would be desirable to diagnose patients with AF at an early stage, when responsiveness for rhythm control therapy is still high.

In contradiction to these limited advances in unravelling of the underlying pathology, the risk for AF-related stroke was considerably lowered by the efficacy of nonvitamin K antagonist oral anticoagulants (NOAC) with tolerable side effects [20] in addition to the established and effective usage for vitamin K antagonists. In case of ESUS, recent approaches to prevent stroke as secondary treatment showed even comparable effects between treatment by NOAC and platelet inhibitors [8], emphasizing the need for a screening of AF in patients with ESUS.

### Cost considerations

To address early diagnosis of SCAF, large-scale AF screening studies have been carried out over the last few years. The majority of these studies applied easy-to-use single-lead ECG devices either by single measurement or multiple measurements within a distinct time frame without testing for cost effectiveness as primary objective. In a few cases, retrospective analysis by Markov analysis and other modelling showed that AF screening was cost effective under particular circumstances and in specific cohorts [2, 15]. However, cost effectiveness strongly depends on AF prevalence in the tested cohort and the available health care system [7]. Unfortunately, cost effectiveness has not yet been tested for AF mass screening in a longitudinal study covering several years of follow-up.

In terms of requirements for successful mass screening approach, recent developments in diagnosis and treatment offer the opportunity for successful implementation, but more evidence is needed to define the patient cohort that will benefit in terms of improved outcome and cost effectiveness. In this review, we will focus on single-lead ECG devices as an approach for AF screening. Implantable devices to determine the heart rhythm such as implantable loop recorders or other approaches to determine heart rhythm [16] are considered for AF screening under specific circumstances in high-risk patients but can for obvious reasons not be seen as a desirable approach for large-scale AF screening.

## Current screening approaches

Since AF screening approaches mainly included patients in specific age groups [6, 15, 22], using certain infrastructure or locations [4, 11, 15, 23–25], cohorts are not necessarily comparable. Regardless of the specific in- and exclusion criteria, patients undergo mainly two different types of screening:

- Opportunistic approach: Patients are screened on appearance once at a single time point measurement
- Systematic approach: Patients are preselected and invited for repeated measurements.

As an exemplary approach for opportunistic screening Chan and Choy [4] investigated the feasibility of mass, territory-wide single time point AF screening. Within 12 months, 13,122 Hong Kong citizens participated, with an AF prevalence of 1.8%. AF was newly diagnosed in 0.8%. The study provided a high number of measurements in an overall low-risk population emphasizing the feasibility of large-scale opportunistic screening. But the impact on diagnosis and treatment remains unclear as there was no confirmation of AF with a 12-lead ECG and no follow-up care was offered. Furthermore, compliance and therapy adherence was not tested. Lack of this information hampers interpretability of this kind of approach for the transfer to routine large-scale AF screening.

As an example for systematic AF screening, the STROKESTOP study invited half of the 75- to 76-year-old population of two regions in Sweden to participate in an ambulatory AF screening program [24]. Within a 28-month inclusion period, 7173 participants (53.8% of all invited patients) were intermittently investigated during a period of two weeks with a handheld single-lead ECG device. Prevalence of AF in this cohort was 9.3% with 3.0% newly detected AF. In total, 5.1% of the screened population had untreated AF. Through repeated measurements the chance of identifying patients with paroxysmal AF was improved [6, 14]. In the STROKESTOP study, the initial ECG recording showed an AF prevalence of 0.5% which increased to 3% through repetitive measurements. Initiation of anticoagulation therapy was accepted by 90% of patients with previously undetected AF but may be biased through preselection since screening was on a voluntary basis. On the basis of the STROKESTOP cohort, the approach was calculated in a model as cost effective [2] but cost effectiveness may be biased by the high age of participants and the associated high prevalence of AF. The inclusion rate of 53.8% is comparable to similar approaches in other studies but still disappointing for the ambition of high coverage, large-scale screening. Additionally, the approach required highly motivated investigators and patients. This is a challenge for cost effectiveness and practicability; thus, the STROKESTOP approach may not be easily implemented in wide-spread daily practice.

Depending on the protocol used in AF screening studies, the rates of newly detected AF varied between 0.8–3% [4, 24], whereas the overall prevalence showed ranged from 3.7–12.3% [11, 24]. Values were higher in studies with multiple ECG measurements and in cohorts with older participants [24]. In contrast, for cohorts with high risk for AF or stroke, implanted devices for continuous monitoring offer a better diagnostic yield. By using implanted devices, annual incidence rates above 20% [9, 19] were measured. These high incidence rates might be related and biased by the preselected cohort but could be also a sign that intensive and continuous monitoring helps to identify episodic SCAF. The majority of these studies used a structured protocol, performing measurements in an environment which was accepted as medical proxy. In particular pharmacies seemed to be accepted by patients and offer an interesting environment through their basic medical supervision, providing reliable measurements.

The deployed noninvasive devices were single-lead ECGs employing an algorithm which mainly interprets RR intervals to detect irregularity and tachycardia as surrogates for AF. Unfortunately, due to technical limitations and a lack of medical supervision during recording, the rate of false-positive measurements remains high which is expected to lower cost effectiveness and acceptance by patients and medical care providers [1].

## Opportunities and challenges of large-scale screening

Thanks to the advances achieved in recent years, the opportunity for large-scale AF screening to detect AF has become possible. New noninvasive screening technologies are in development and have already been part of mass screening programs on an investigative level with promising results. NOACs offer effective and safe therapy once AF is diagnosed with higher compliance in treated patients and a lower rate of intracranial bleedings compared to previous anticoagulant therapy. An increasing incidence of AF is expected due to ageing of our population and recent evidence suggests that the prevalence is underestimated because of the large percentage of asymptomatic AF episodes. Thanks to educational efforts, a higher awareness of health-related issues in the population correlates with a concomitant higher demand in medical care. Through the ubiquitous availability of sensors integrated into smartphones, apps, and wearables, biological measurements are provided by nonmedical companies. The availability of sensitive sensors and improvements in patient awareness regarding their health have facilitated the spread of paramedic diagnostic tools for AF detection and offer the opportunity for more opportunistic screening in our population.

There remain challenges which have to be handled for reliable and effective results in the diagnosis and treatment of AF. The diagnostic accuracy of available technology like single-lead ECG, biomarkers, and risk scores is unacceptably low. Therefore, at the moment, they may only allow to rule-out current AF but are not sufficiently robust to confirm the diagnosis of AF without a standard ECG recording.

Once AF is diagnosed, structured care pathways have to be defined [22] and therapy adherence must be ensured [18, 21, 24]. From the available literature, there is good evidence for the feasibility of large-scale AF screening but little is known about the follow-up, confirmation of AF, and—in particular—the efficacy of initiated anticoagulant therapy to reduce morbidity and mortality. Because large-scale AF screening may lead to the broader use of anticoagulant therapy, possible positive or negative effects on outcome have to be established in future studies. Also, the cost effectiveness of such an approach has to be addressed. This question is complicated by the fact that cost effectiveness of AF screening likely depends on local circumstances and reimbursement strategy of the national health care systems. Therefore, no generally valid cost effectiveness may be calculated. Certainly, following structured approaches similar to the STROKESTOP study [2], large-scale AF screening focusing on high-risk patients has the highest chance to be cost effective and improve therapy adherence [2, 15].

## Ideal large-scale AF screening

Large-scale AF screening is desirable but should only be carried out in well-defined populations. The cohort of patients at risk for AF who will benefit from screening and treatment has to be identified in upcoming randomized trials. Evidence suggests that patients of 65 years or older, suffering subclinical or atypical symptoms of AF, severe cardiovascular disease (previous other atrial arrhythmia, heart attack, moderate cardiomyopathy or valve disease), or a CHA_2_DS_2_Vasc score of 2 or higher might benefit from AF screening. For patients 65 years or older, guidelines recommend opportunistic screening by taking the pulse [12] at each general practitioner visit.

**Fig. 1:**
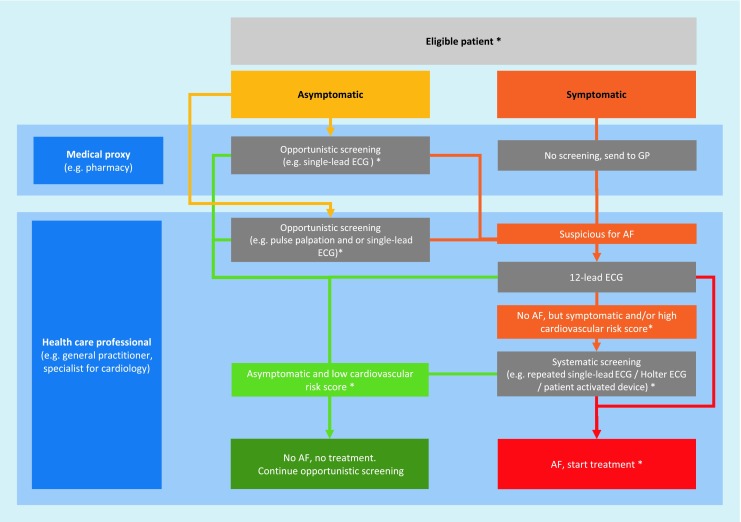
Screening for atrial fibrillation (AF) patients. Asymptomatic eligible patients should be screened in an opportunistic way. Symptomatic patients or patients with suspicion of AF should receive a systematic screening to diagnose or exclude AF. Cohorts, diagnosis of AF, and treatment marked with an *asterisk* (*) have to be defined in upcoming randomized control trials

For future AF screening, we propose to broaden screening opportunities with the help of single-lead ECG devices at each GP visit in addition to or instead of palpatory pulse control (Fig. 1). In cases of suspected AF, a handheld ECG device with at least one lead should be given to patients for daily measurement for at least one month and in case of symptoms. To confirm diagnosis of AF, at least one episode of 30 s in one lead of invasive or noninvasive ECG should be present. Furthermore, confirmation of AF must be made by a physician and not by automated analysis.

Sensors or smartphone apps that are not approved for AF diagnosis may identify patients at risk of AF or with suspicion of AF but initiation of AF screening by an ECG with at least one lead must be at the discretion of the treating physician. Biomarker assays may help in future attempts to rule out AF or may initiate further screening by ECG but no preventive treatment like anticoagulation may be initiated without ECG confirmation of AF.

## Practical conclusion

- Large-scale AF screening may offer improvements for early detection of AF and may result in intensified monitoring of cardiovascular disease in tested patients, but should only be carried out in well-defined at-risk cohorts.
- New hand-held ECG devices already provide promising results but require further improvement.
- Diagnosis of AF requires that the AF episode be recorded by an ECG device.
- Respective clinical trials are currently clarifying the role of atrial high rate episodes, subclinical and symptomatic AF in terms of risk of stroke and pathway of care.
- A pathway of care for patients screened for AF has to be defined.
- Whether anticoagulant therapy should be initiated in patients with AF detected in larger screening studies remains unanswered.
- Longitudinal randomized control trials in terms of clinical outcome and cost effectiveness are mandatory to determine the possibilities of large-scale AF screening.

## Abbreviations

AF: Atrial fibrillation
ESUS: Embolic stroke of unknown source
NOAC: Nonvitamin K antagonist oral anticoagulants
SCAF: Subclinical atrial fibrillation
